# Acute HIV Infection Presenting With Diffuse Plaque Psoriasis Treated With Highly Active Antiretroviral Therapy

**DOI:** 10.7759/cureus.19680

**Published:** 2021-11-17

**Authors:** Moshe Y Bressler, Naeha Pathak, David Rotblat, Rebecca Tamez

**Affiliations:** 1 Dermatology, New York Institute of Technology College of Osteopathic Medicine, Old Westbury, USA; 2 Dermatology, Icahn School of Medicine at Mount Sinai, New York, USA; 3 Internal Medicine, Staten Island University Hospital, Staten Island, USA; 4 Dermatology, Jamaica Hospital Medical Center, Queens, USA

**Keywords:** acute retroviral syndrome, viral exanthem, diffuse psoriasis, hiv symptoms, haart, acute hiv infection

## Abstract

Cutaneous diseases such as psoriasis are often the first disease manifestations in HIV+ patients, with greater severity corresponding to a weaker immune system. Despite its prevalence, literature and placebo-controlled studies on the recognition of HIV as a cause of psoriasis are lacking, causing challenges to arise in its treatment. In this article, we illustrate a case of an HIV+ patient whose psoriasis drastically improved after the initiation of highly active antiretroviral therapy (HAART) consisting of bictegravir, emtricitabine, and tenofovir alafenamide. While it is unclear which combination of antiretrovirals is optimal for controlling psoriasis in HIV+ patients, prompt initiation of HAART can significantly improve immune status and psoriasis in HIV+ patients.

## Introduction

HIV is commonly associated with skin diseases, particularly psoriasis. The prevalence of psoriasis in HIV patients is similar to the general population, at 2-3% [1]. The onset of psoriasis in patients with HIV is more severe [2], corresponding to a low CD4+ count and an overall decreased immune status [3], and poses challenges in diagnosis and treatment. Managing psoriasis in HIV+ patients presents unique challenges; traditional immunosuppressants may increase susceptibility to infections and can interact with antiretroviral therapy [4]. In this paper, we discuss a case of a patient with HIV whose psoriasis drastically improved after using highly active antiretroviral therapy (HAART) (bictegravir, emtricitabine, and tenofovir alafenamide), indicating that early initiation of HAART with this medication combination can improve HIV and psoriasis in patients.

## Case presentation

**Figure 1:**
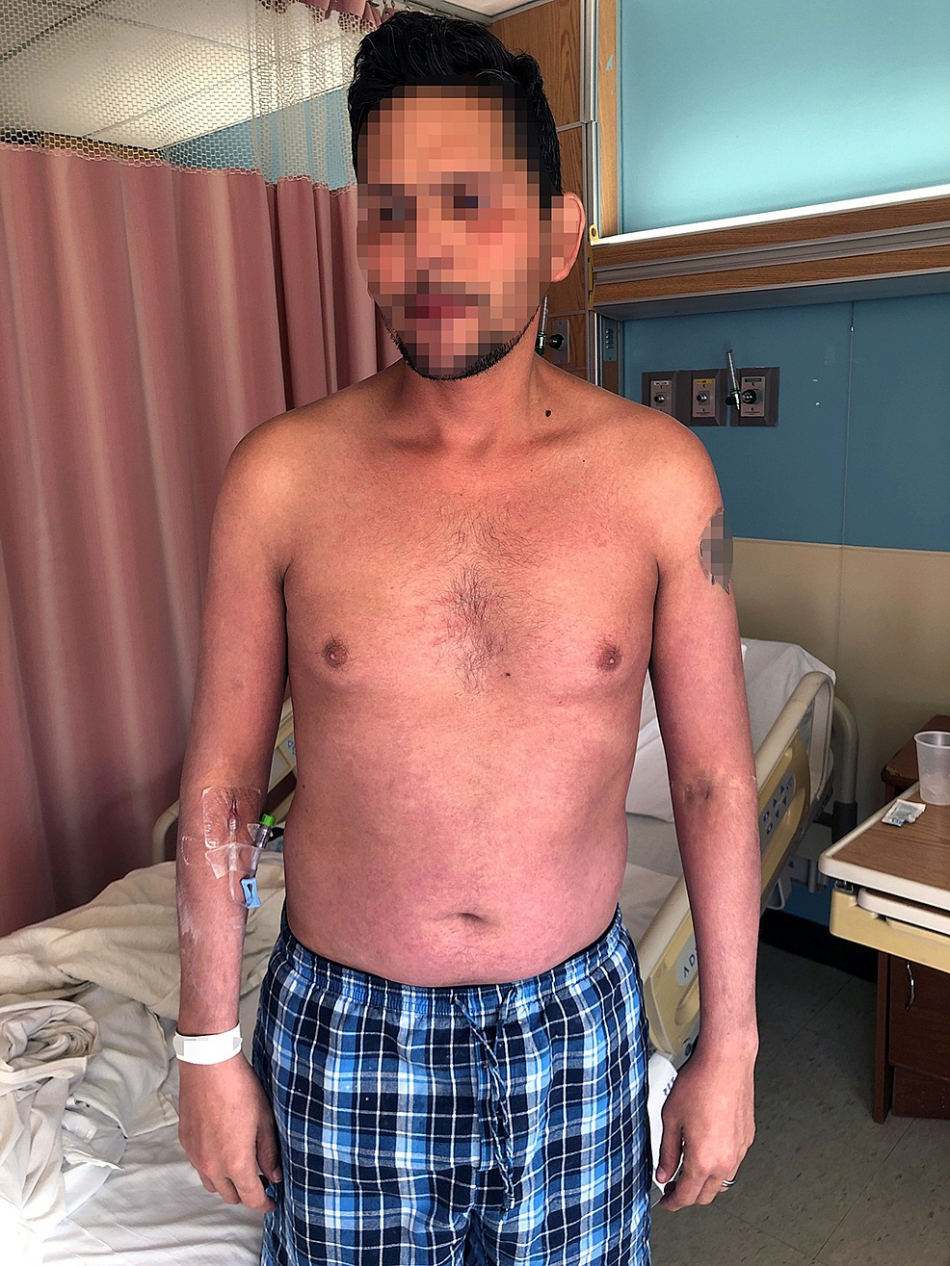
New erythematous maculopapular rash on the face, bilateral arms, chest, and abdomen.

**Figure 2:**
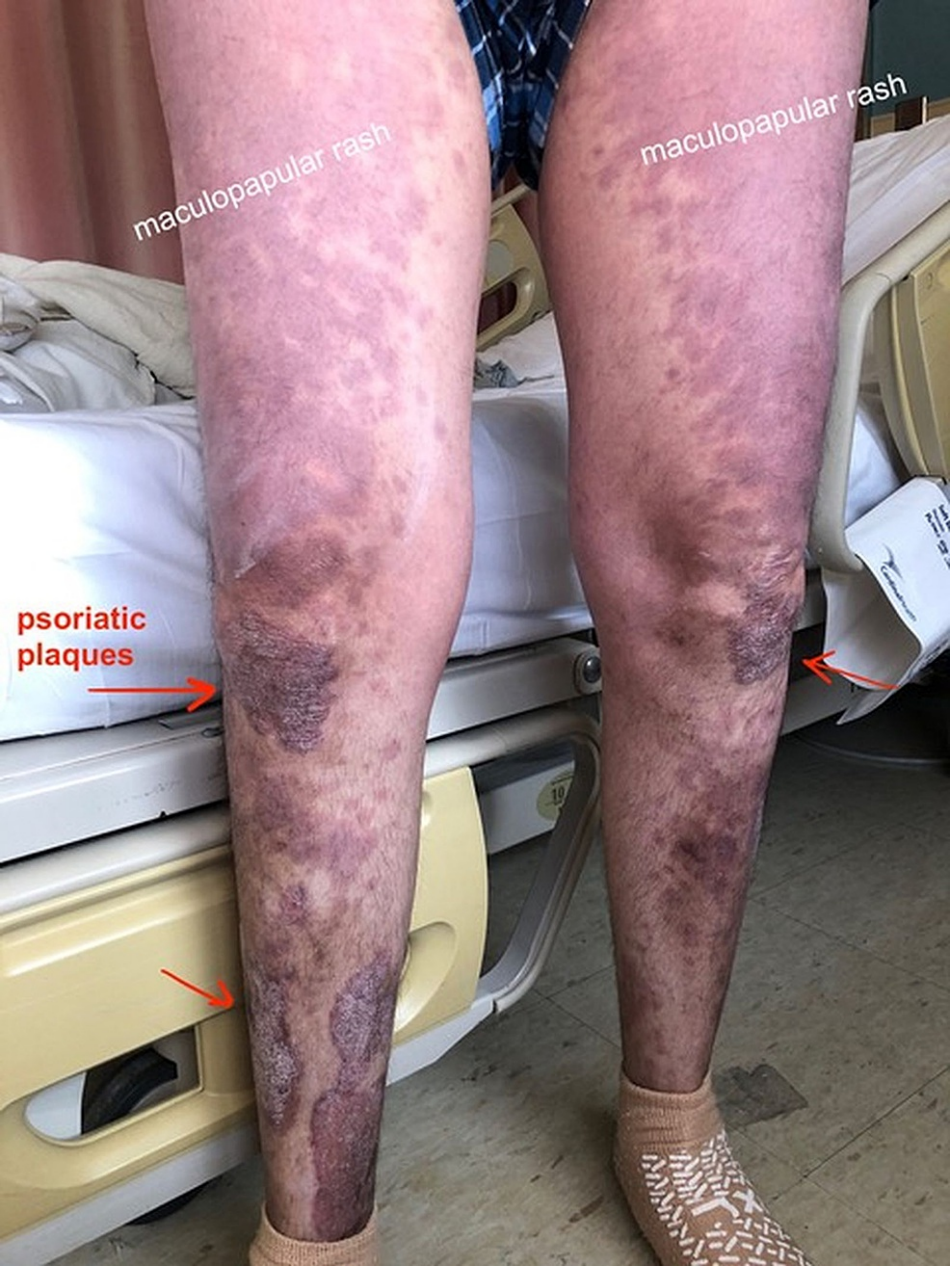
Maculopapular rash on the lower extremities with acutely worsening plaque psoriasis.

A 39-year-old Latino male with a history of psoriasis presented to the emergency room with fever for three days and a new-onset pruritic generalized maculopapular rash on his face, torso, and proximal extremities (Figure 1). Additionally, he was found to have acutely worsening plaque psoriasis on his legs and arms (Figure 2). He had started no new medications and denied any new exposures or sick contacts. Upon further history, he shared that he was sexually active and an intermittent IV drug user. He was otherwise healthy and had no other significant past medication history.

Punch biopsies confirmed a dermal hypersensitivity reaction of the thigh and plaque psoriasis of the shin. Further evaluation revealed acute HIV infection with a CD4+ count of 10/μl and viral load of 14,700 copies/mL. The clinical picture of the combination of acute onset dermal hypersensitivity eruption, pruritus, fever, and worsening psoriasis was consistent with the acute retroviral syndrome (ARS). The patient was started on topical corticosteroids and daily antiretroviral therapy with combined bictegravir 50 mg, emtricitabine 200 mg, and tenofovir alafenamide 25 mg (Biktarvy). Antibacterial prophylaxis was initiated with daily sulfamethoxazole 800 mg and trimethoprim 160 mg (Bactrim DS) and azithromycin 1200 mg weekly. At one month follow-up after discharge, the acute HIV rash was found to be resolved and the plaque psoriasis greatly improved with only minor involvement remaining over bilateral knees.

## Discussion

Some common manifestations of HIV include infectious diseases, cancer, and rheumatic diseases [4]. In particular, cutaneous diseases are a common manifestation of HIV, similar to that of the general population, that often results in higher morbidity and mortality [1,5]. Sudden onset psoriasis or abrupt worsening of psoriasis may be an indication of ARS and require HIV testing [6]. Psoriasis is an autoimmune disease that is thought to be reliant on an intact immune system; however, when there is a decline in immune status, as in our patient with acquired immunodeficiency syndrome (AIDS), psoriasis worsens. This is thought to be due to the decrease in regulatory T cells, particularly the imbalance of CD4+ and CD8+ cells, causing dysregulation toward self-antigens, increasing cytokine production, and autoimmune reactivity [6]. This may also play a role in the pruritic erythematous exanthem caused by ARS [6]. Of note, our patient’s new diffuse rashes, as well as worsening psoriatic plaques, were the sole reason this patient sought out treatment, suggesting that HIV testing should be considered in similar cases.

Treatment of psoriasis in HIV+ patients can be challenging and refractory cases should be treated in conjunction with an infectious disease specialist [6]. Due to the lack of randomized placebo-controlled studies, the National Psoriasis Foundation recommended treatments are primarily based upon case reports and case series [5]. Newly diagnosed HIV should be treated with antiretrovirals, and usually, a rise in CD4+ count tends to improve psoriasis [7]. Mild to moderate cases can be treated with topical therapy, and moderate to severe cases with phototherapy or oral retinoids [8]. Unresolved cases have been reportedly treated with methotrexate, cyclosporine, tumor necrosis factor inhibitors, and hydroxyurea [6]. Additionally, biologics targeting interleukin-23 (IL-23) may be a favorable option in HIV since they are less likely to interact with opportunistic infections [9]. These immunosuppressants require close monitoring of CD4+ count, opportunistic infections, and malignancies [5].

## Conclusions

It is important to recognize HIV as a potential cause for severe skin conditions, as early intervention can drastically improve patient outcomes. Acute retroviral syndrome (ARS) presents with generalized maculopapular rash, constitutional symptoms, cutaneous changes, and a paradoxical worsening of immune-mediated conditions such as psoriasis, an autoimmune disease that depends on an intact immune system. Thus, as immune status decreases with AIDS, the severity of psoriasis increases. Our study shows the patient’s psoriasis completely resolving upon initiation of HAART using bictegravir, emtricitabine, and tenofovir alafenamide. Furthermore, the clinical photographs from this case can help raise awareness of the presentation of psoriasis in HIV+ individuals with skin of color.
